# Development of a Multifunctional Oral Dosage Form via Integration of Solid Dispersion Technology with a Black Seed Oil-Based Self-Nanoemulsifying Drug Delivery System

**DOI:** 10.3390/biomedicines11102733

**Published:** 2023-10-09

**Authors:** Abdelrahman Y. Sherif, Ahmad Abdul-Wahhab Shahba

**Affiliations:** 1 Department of Pharmaceutics, College of Pharmacy, King Saud University, P.O. Box 2457, Riyadh 1145, Saudi Arabia; ashreef@ksu.edu.sa; 2 Kayyali Research Chair for Pharmaceutical Industries, College of Pharmacy, King Saud University, P.O. Box 2457, Riyadh 11451, Saudi Arabia

**Keywords:** lansoprazole, solid dispersion, bioactive SNEDDS, black seed oil, multifunctional drug delivery systems

## Abstract

Lansoprazole (LZP) is used to treat acid-related gastrointestinal disorders; however, its low aqueous solubility limits its oral absorption. Black seed oil (BSO) has gastroprotective effects, making it a promising addition to gastric treatment regimens. The present study aims to develop a stable multifunctional formulation integrating solid dispersion (SD) technology with a bioactive self-nanoemulsifying drug delivery system (SNEDDS) based on BSO to synergistically enhance LZP delivery and therapeutic effects. The LZP-loaded SNEDDS was prepared using BSO, Transcutol P, and Kolliphor EL. SDs were produced by microwave irradiation and lyophilization using different polymers. The formulations were characterized by particle apparent hydrodynamic radius analysis, zeta potential, SEM, DSC, PXRD, and in vitro dissolution testing. Their chemical and physical stability under accelerated conditions was also examined. Physicochemical characterization revealed that the dispersed systems were in the nanosize range (<500 nm). DSC and PXRD studies revealed that lyophilization more potently disrupted LZP crystallinity versus microwave heating. The SNEDDS effectively solubilized LZP but degraded completely within 1 day. Lyophilized SDs with Pluronic F-127 demonstrated the highest LZP dissolution efficiency (3.5-fold vs. drug) and maintained chemical stability (>97%) for 1 month. SDs combined with the SNEDDS had variable effects suggesting that the synergistic benefits were dependent on the formulation and preparation method. Lyophilized LZP-Pluronic F127 SD enabled effective and stable LZP delivery alongside the bioactive effects of the BSO-based SNEDDS. This multifunctional system is a promising candidate with the potential for optimized gastrointestinal delivery of LZP and bioactive components.

## 1. Introduction

Gastrointestinal disorders associated with gastric hyperacidity, such as peptic ulcers and gastroesophageal reflux disease (GERD), are prevalent conditions that can be exacerbated by factors including *Helicobacter pylori* infection, non-steroidal anti-inflammatory drug (NSAID) use, corticosteroid administration, alcohol consumption, stress conditions, and continuous intake of spicy and caffeine-containing products [1,2,3,4].

**Figure 1:**
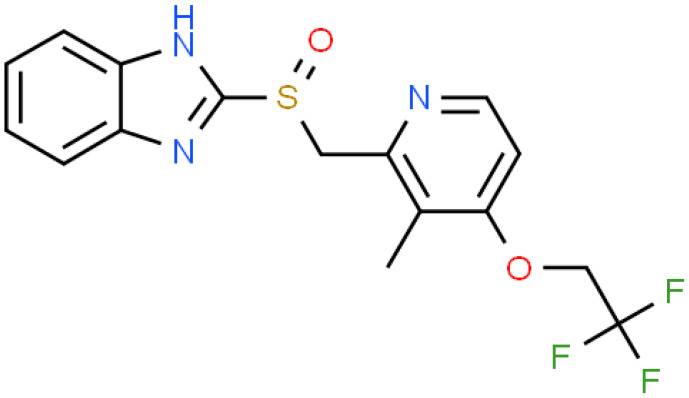
The chemical structure of lansoprazole (obtained from ChemSpider chemical structure database, http://www.chemspider.com/, accessed on 28 September 2023).

Proton pump inhibitors (PPIs) like lansoprazole (Figure 1) are commonly prescribed to reduce gastric acid production through inhibition of the H+/K+-ATPase enzyme with a typical daily dosage of 15–30 mg [5]. However, lansoprazole has low aqueous solubility and is classified as a Biopharmaceutics Classification System Class II drug, resulting in incomplete dissolution from oral dosage forms, and low and erratic oral bioavailability [6,7]. Various formulation strategies were conducted to improve solubility, dissolution, and absorption of LZP. For example, Zhang et al. prepared an SD formulation of LZP using fluid-bed coating technology where 90% of drug was dissolved at the end of experiment [8]. In alignment with such an achievement, various studies showed that SD formulations were able to enhance drug bioavailability compared to pure LZP [9,10]. Moreover, Ubgade et al. prepared a nanosuspension formulation of LZP and in vitro dissolution showed that the prepared formulation was able to enhance initial and total drug dissolution behavior compared to the pure drug [11].

Self-nanoemulsifying drug delivery systems (SNEDDSs) incorporate oils, surfactants, and co-surfactants to form nano-scale emulsion droplets upon mild agitation, enhancing the surface area for dissolution and absorption compared to the drug alone [12]. Recent research incorporated naturally derived oils with biological activities as an attempt to potentially augment the therapeutic activity of administered therapeutic molecules [13].

In light of this, several reports revealed that black seed oil (BSO) has a protective and healing effect on gastric ulcers [14,15]. Along with this, various studies showed that the administration of thymoquinone (TMQ), the major constituent in BSO, reduces peptic ulcers produced by NSAIDs and other agents like ethanol [16,17,18]. In detail, Kanter et al. studied the impact of BSO and TMQ on gastric ulcers in rats. Their study reported that both BSO and TMQ were able to reduce peptic ulcer indices, with a more prominent reduction observed in the BSO-treated group [14]. This suggests that the incorporation of BSO rather than TMQ alone into SNEDDS formulations could combine the benefits of TMQ alongside additional protective components intrinsic to BSO. Furthermore, Radwan et al. prepared a TMQ-loaded SNEDDS to study the impact of increasing drug solubility on the therapeutic effects. Their study reported that the formulated TMQ-SNEDDS decreased the ulcer index by 2-fold compared to free TMQ alone, as indicated in their findings [17]. Despite their reported in vivo activity, the FDA has not yet approved black seed extracts or concentrated thymoquinone for treating any medical conditions like high cholesterol, diabetes, or high blood pressure. However, black seed extracts remain available for purchase over-the-counter as dietary supplements marketed to aid digestion and promote energy levels. Research has consistently shown black seed derivatives to be well-tolerated and safe, as evidenced by their designation as “generally recognized as safe” (GRAS) by the FDA [19].

While the formulation of drugs within SNEDDSs addresses solubility limitations, practical challenges with physical/chemical instability over the shelf-life still restrict clinical translation. Chemical instability might arise from incompatibilities between excipients and the loaded drug, leading to its degradation in the presence of these excipients [20]. Thus, formulated SNEDDSs are often separated from the therapeutic agent to circumvent stability issues [21,22]. As an alternative technology, SDs represent stabilized carrier systems capable of maintaining improved drug performance through nano-dispersed or molecularly dissolved drug domains within hydrophilic polymer matrices [23].

The current research proposes a novel formulation approach combining solubility- and stability-enhancing technologies with a bioactive natural oil into a single optimized product. The specific aims are to prepare and characterize drug-free and drug-loaded BSO-SNEDDSs; fabricate lansoprazole SDs and evaluate their in vitro dissolution; assess the effects of BSO-SNEDDSs on SD dissolution behavior; and conduct stability testing to identify a lead candidate formulation. The findings could provide clinically translatable dosage forms with synergistic delivery of lansoprazole and bioactive BSO components for a potentially more effective treatment for acid-related gastrointestinal disorders.

## 2. Materials and Methods

### 2.1. Procurement of Plant Material and Isolation of Bioactive Components

The methodologies implemented for assembling, isolating, and calibrating black seed oil (BSO) have been delineated comprehensively within our prior investigative studies [13,24,25].

### 2.2. Chemical and Reagents

The proton pump inhibitor lansoprazole was acquired from Mesochem Technology (Beijing, China). The surfactant Kolliphor EL (KrEL) was obtained from BASF (Ludwigshafen, Germany). The triblock copolymer Pluronic-F127 (PF-127) was sourced from Sigma Aldrich (St. Louis, MO, USA). The co-solvent Transcutol^®^ P (TCP) was provided by Gattefossé (Lyon, France). The polymer polyethylene glycol 4000 (PEG 4000) was procured from BDH Chemicals Ltd. (Poole, UK). The gelatin capsules size 0 were supplied by Capsugel (Morristown, NJ, USA). The cellulose derivative hydroxypropyl methylcellulose (HPMC) E3 was acquired from JRS Pharma (Rosenberg, Germany).

### 2.3. Preparation and Characterization of LZP-SNEDDS

Self-nanoemulsifying drug delivery systems (SNEDDSs) were developed utilizing black seed oil, Transcutol P, and Kolliphor EL at optimized concentration ratios (25/25/50 *w*/*w*). For the preparation of a drug-free SNEDDS, the components (2 g) were added to vials in the specified amounts and blended using a vortex mixer. Drug-loaded SNEDDSs were formed by incorporating lansoprazole (30 mg) into the formulation ingredients using a similar procedure. Both drug-free and drug-loaded SNEDDSs, with and without accompanying solid dispersions, were subjected to dilution with deionized water (at a 1:1000 *w*/*w* ratio) and then subjected to mixing for 1 min. The resulting solutions were centrifuged and analyzed using a Zetasizer dynamic light scattering instrument (Model ZEN3600, Malvern Instruments Co., Worcestershire, UK) to determine the particle apparent hydrodynamic radius and zeta potential of the dispersed systems. This enabled the characterization of the nanoemulsion properties formed upon dilution of the self-emulsifying formulations [25].

### 2.4. Preparation of Solid Dispersion (SD) Formulation

Solid dispersions (SDs) of lansoprazole (LZP) were prepared in this work using microwave irradiation (MW) and lyophilization (LP) techniques. The polymers Pluronic F-127 and polyethylene glycol 4000 (PEG4000) were selected for MW preparation due to their relatively low melting points, which enabled uniform heating and mixing during irradiation. In contrast, Pluronic F-127 and hydroxypropyl methylcellulose (HPMC) were used for LP preparation. The compositions of the LZP/polymer ratios and their corresponding preparation methods are outlined in Table 1.

#### 2.4.1. Microwave Method

Lansoprazole (LZP) was blended with the polymers Pluronic F-127 and polyethylene glycol 4000 (PEG4000) in a 1:4 ratio by weight to produce mixtures for microwave solid dispersions (MW-SDs) [26]. Approximately 1 g of each drug–polymer mixture was thoroughly mixed in a porcelain mortar to achieve a homogeneous preparation. Domestic microwave irradiation (Samsung Model ME0113M1) was then utilized to prepare the MW-SDs [21,27]. The microwave instrument was preheated for around 2 min prior to irradiation of the mixtures. The LZP-polymer mixtures were subjected to microwave radiation at 900 W power for about 2 min and 6 min to obtain the MW-PF-127 and MW-PEG-4000 MW-SD formulations, respectively. The molten dispersions were stirred continuously with a glass rod during irradiation to maintain homogeneity. Upon cooling to room temperature, the solidified dispersions were gently crushed and sieved through a 315 μm screen to achieve uniform fine powders.

#### 2.4.2. Lyophilization Method

A preliminary LZP solubility study was conducted to select the optimum pH for preparing the LZP solution. Among the three tested pH levels (9.2, 10.0, and 10.8), the latter showed the highest LZP solubility (1.882 ± 0.069 mg/mL) and therefore, was selected as the optimum pH for the lyophilization process. LZP was dissolved in the prepared bicarbonate buffer (pH 10.8) using a magnetic stirrer to generate an ~0.74 mg/mL solution. Predetermined quantities of the polymers Pluronic F-127 and hydroxypropyl methylcellulose (HPMC) were added to the LZP solution at a 4:1 ratio by weight and mixed thoroughly to produce formulations for lyophilized solid dispersions (LP-SDs). The prepared drug–polymer solutions were frozen at −60 °C prior to lyophilization. The frozen dispersions were then lyophilized for at least 48 h at −60 °C using a freeze dryer (Al-pha 1-4 LD Plus, Osterode am Harz, Germany) to allow solvent sublimation. This could potentially achieve a porous matrix with the drug molecularly dispersed in the polymer scaffold. The obtained LP-SDs were gently crushed and sieved through a 315 μm screen to achieve uniform fine powders [28].

### 2.5. Scanning Electron Microscopy (SEM)

The prepared microwave solid dispersions with Pluronic F-127 and PEG4000 (MW-PF-127 and MW-PEG-4000) and the lyophilized solid dispersions with Pluronic F-127 and HPMC (LP-PF-127 and LP-HPMC) were analyzed using scanning electron microscopy (SEM). The samples were mounted on stubs and sputter coated with gold for 60 s at 20 mA using a Q150R sputter coating unit (Quorum Technologies Ltd., East Sussex, UK) under an argon atmosphere. This allowed examination of the surface morphology and topography of the different solid dispersion formulations using SEM imaging (Carl Zeiss EVO LS10, Cambridge, MA, USA) under high vacuum [24,28].

### 2.6. Differential Scanning Calorimetry (DSC)

Differential scanning calorimetry (DSC) was utilized to characterize the prepared solid dispersion samples using a DSC-60 instrument (Shimadzu, Kyoto, Japan). Approximately 2 mg of each sample was weighed into a non-hermetically sealed aluminum pan. The samples were heated from 25 °C to 250 °C at a 10 °C/minute heating rate to obtain thermographs. The DSC measurements were performed under a nitrogen atmosphere with a 40 mL/min flow rate. Post-analysis, the DSC curves were subjected to baseline manual correction using TA 60 thermal analysis software. This enabled investigation of the thermal behavior and identification of any thermal events such as melting, crystallization, or degradation in the solid dispersion formulations [29].

### 2.7. Powder X-ray Powder Diffraction (PXRD)

Powder X-ray diffraction analysis was conducted to investigate crystallinity changes after SD preparation. The LZP, polymers, physical mixtures, and SD formulations were subjected to an X-ray diffractometer instrument (Ultima IV, Rigaku Inc. Tokyo, Japan). The obtained PXRD pattern was investigated to assess the crystalline state of LZP within the prepared SD formulations. Each sample was measured in the scanning range of 3–60° with a scanning rate of 1°/min using an X-ray diffractometer. The characteristic peak of each sample was assessed by collecting the data by monochromatic radiation (Cu Kα’ 1, λ = 1.54 Å), operating at a voltage of 40 kV and current of 40 mA. This allowed evaluation of the nature of crystallinity of lansoprazole within the solid dispersion formulations compared to pure drug and physical mixtures [28].

### 2.8. In Vitro Dissolution Test

Dissolution tests were performed to evaluate and compare the drug release behavior of formulations. A USP Type II dissolution testing apparatus (UDT-814, LOGAN Inst. Corp., Franklin, NJ, USA) was employed for the study. Formulations containing equivalent amounts (15 mg) of lansoprazole were placed in capsules, surrounded by sinkers, and placed into vessels containing 900 mL of pH 6.8 phosphate buffer (prepared according to European pharmacopeia specifications). Paddles were set to rotate at 75 rpm for the duration of the dissolution experiments. Prior to initiating the release studies, buffer media were equilibrated to 37 °C in the jacketed vessels. Samples were manually drawn from vessels at pre-defined time points of 5, 10, 15, 30, 60, and 120 min. An in-line filter assembly was used to withdraw aliquots, which were subsequently analyzed by a validated UPLC method to determine the amount of drug dissolved over time [28]. Formulation performance was compared based on the dissolution efficiency (DE)% [21,30].

### 2.9. Stability Study

The thermal stability of SD, drug-loaded SNEDDS, and raw LZP drug substances was evaluated under accelerated conditions. All samples were packaged in tightly capped amber glass containers to protect them from moisture and light exposure. These stability test units were then placed in programmable climate-controlled chambers (Binder GmbH, Tuttlingen, Germany) preset to maintain 40 ± 2 °C and 75 ± 5% relative humidity. At time points of 1, 7, and 30 days, samples were retrieved and their drug content was analyzed using the UHPLC analytical method. This experiment was designed to assess and compare the changes in lansoprazole content and overall stability of the various pharmaceutical systems exposed to elevated heat and moisture over time [31].

### 2.10. Quantification of LZP Using the Developed UPLC-UV Method

Lansoprazole (LZP) was quantified using the ultra-high performance liquid chromatography with ultraviolet detection (UHPLC-UV) method. The analyses were performed on a Dionex UltiMate 3000 UHPLC system equipped with an autosampler and DAD detector (Thermo Scientific, Bedford, MA, USA). An Acquity BEH C18 column (2.1 × 50 mm, 1.7 μm) was used for separation. The column temperature was maintained at 40 ± 0.5 °C. An isocratic mobile phase consisting of 0.1% triethylamine (pH 6.7)/acetonitrile (58:42, *v*/*v*) achieved separation of LZP. The flow rate was 0.4 mL/min and the detection wavelength was set at 320 nm.

### 2.11. Software

The data from the current study were analyzed primarily using the Python programming language (version 3.9.13) within a Jupyter Notebook environment. The specific Python packages utilized included NumPy for data manipulation, Pandas for data frames, Matplotlib and Seaborn for visualization, StatAnnotations for statistical validation, and itertools for additional data processing functions. Portions of this article describing the data analysis methods and presenting results were composed with writing assistance from Claude (developed by Anthropic and operated by Poe) and Bing AI chat. Some Python scripts supporting the data analysis were also developed with input from these AI tools. However, the authors maintained overall responsibility for the direction, ideas, content, and finalization of the manuscript.

### 2.12. Statistical Analysis

The normality of the data was assessed by the Shapiro–Wilk test (Scipy.stats python package) [32]. The homogeneity of the variances was assessed by Levene’s test (Scipy.stats python package) and homoscedasticity (pingouin python package). For dependent variables (with a fairly normal distribution and equal variances), the independent T-test was used to test the statistical significance between two independent samples, the dependent T-test (Scipy.stats and statannotations python package) for two paired samples, and one-way ANOVA followed by the Tukey’s post hoc test (Scipy.stats and scikit_posthocs python packages) for >2 samples. If the sample contain significant outliers and/or if the normality assumption is significantly violated, Kruskal–Wallis H followed by the Dunn post hoc test with Bonferroni correction (Pingouin and scikit_posthocs python packages) was used for >2 samples [33]. The two-way ANOVA test was carried out to analyze the effect of two independent factors on one dependent variable (OLS and SM from Statsmodels.formula.api and Statsmodels.api python packages, respectively). A *p*-value of ≤0.05 was denoted statistically significant in all the statistical analysis tests.

## 3. Results

### 3.1. Characterization of Lansoprazole-Loaded Self-Nanoemulsifying Delivery System

**Figure 2:**
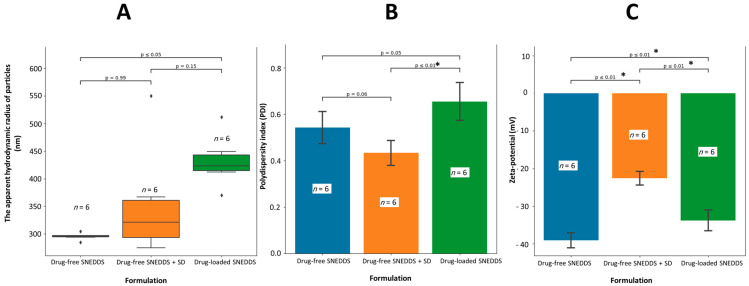
Influence of SNEDDS loading and combination with SD on (**A**) particle apparent hydrodynamic radius, (**B**) PDI (polydispersity index), and (**C**) ZP (zeta potential). SNEDDS (self-emulsifying drug delivery system), and SD (solid dispersion prepared using Pluronic-F127 by lyophilization method). The PS data were statistically analyzed by Kruskal–Wallis H followed by the Dunn post hoc test (Bonferroni correction) while the PDI and ZP data were analyzed by ANOVA followed by Tukey’s post hoc test. A significant *p*-value (<0.05) is marked with an asterisk (*). Outliers in the dataset were symbolized using a diamond shape (◆).

The apparent hydrodynamic radius of the drug-free SNEDDS formulation droplets significantly increased from 295 nm to 431.5 nm after incorporating LZP (*p* < 0.05) (Figure 2A). However, there was no significant difference in the apparent hydrodynamic radius when an SD was added to the SNEDDS formulation. Interestingly, the PDI of the drug-loaded SNEDDS showed a significant increase compared to the combination of the drug-free SNEDDS and SD (*p* < 0.05) (Figure 2B). Additionally, all zeta potential values exhibited significant differences (*p* < 0.05), with the drug-free SNEDDS having the highest value (−39 mV), while the combination with SD had the lowest zeta potential value (−23 mV) (Figure 2C).

### 3.2. SEM

#### 3.2.1. Microwave Method

**Figure 3:**
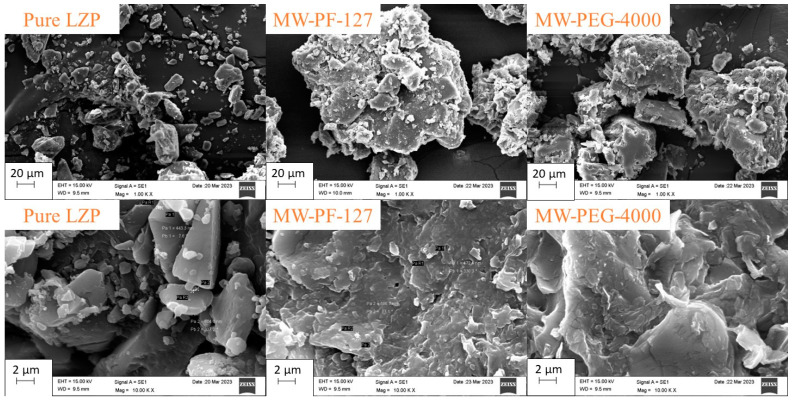
SEM images of pure LZP and MW-SD formulations (MW-PF-127 and MW-PEG-4000).

Scanning electron micrographs revealed that raw lansoprazole exists as small crystals with well-defined edges, indicating its crystalline nature (Figure 3). In contrast, the MW-SD formulations appeared as larger particles with smoother surfaces. This change in morphology suggests that the drug was dispersed within the carrier matrices rather than remaining in a crystalline state. No evidence of phase separation or incomplete solidification was observed in the solid dispersion systems.

#### 3.2.2. Lyophilization Method

**Figure 4:**
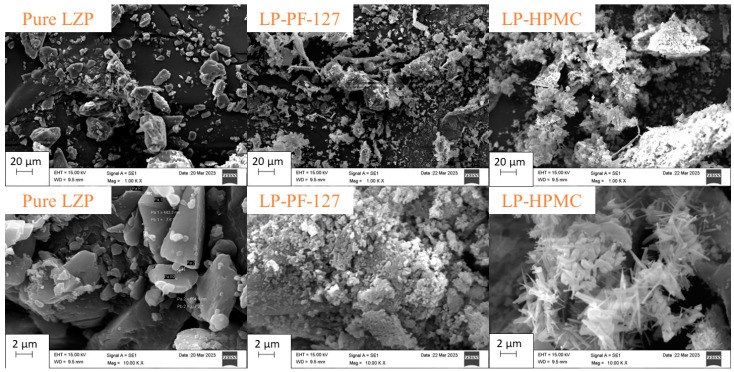
SEM images of pure LZP and prepared LP-SD formulations (LP-PF-127 and LP-HPMC).

Comparison of the scanning electron micrographs revealed noticeable differences in particle size between the microwave (MW) and lyophilized (LP) solid dispersions (SDs). The LP-SD particles prepared with Pluronic F-127 appeared remarkably smaller than their MW-SD counterparts (Figure 4). Additionally, images of the LP-SD with hydroxypropyl methylcellulose (LP-HPMC) showed needle-shaped crystalline structures at higher magnification (Figure 4, LP-HPMC). These may be indicative of incomplete amorphization in the LP-HPMC formulation. Similar to the MW-SDs, no evidence of incomplete separation or residual solvents was observed for the LP-SDs.

### 3.3. DSC

#### 3.3.1. Microwave Method

**Figure 5:**
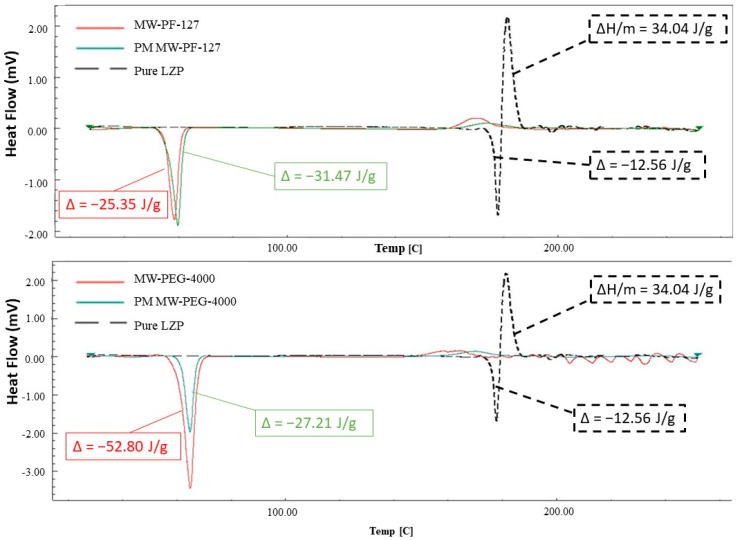
DSC spectra of pure LZP and MW-SD formulations (MW PF-127 and MW-PEG-4000) along with their corresponding physical mixture.

The DSC thermogram of pure lansoprazole (LZP) exhibited a sharp endothermic melting point peak at approximately 178 °C, followed by a decomposition exotherm at around 182 °C (Figure 5). In contrast, the microwave solid dispersion (MW-SD) with Pluronic F-127 and its corresponding physical mixture displayed sharp endothermic peaks at about 58–60 °C. Similarly, the MW-SD with PEG-4000 and its physical mixture showed endotherms at approximately 64 °C. Notably, the characteristic LZP melting and decomposition peaks were completely absent in the thermograms of the MW-SD formulations.

#### 3.3.2. Lyophilization Method

**Figure 6:**
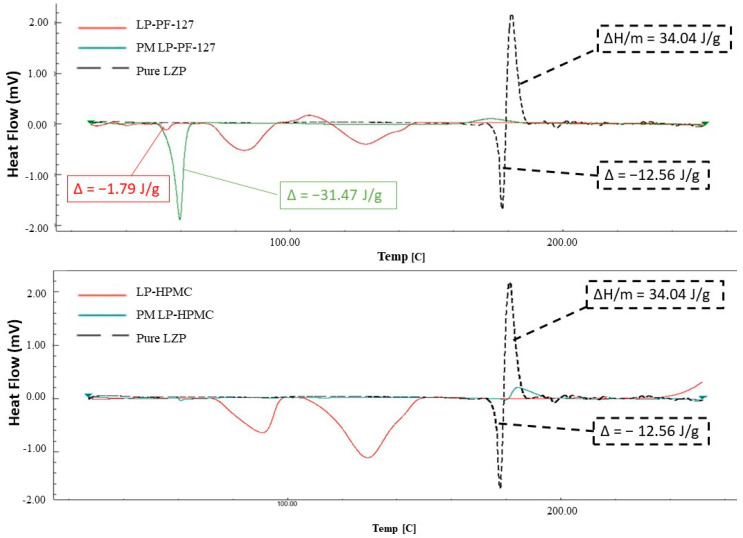
DSC spectra of pure LZP and LP-SD formulations (LP-PF-127 and LP-HPMC) along with their corresponding physical mixture.

The lyophilized formulations also exhibited changes in thermal behavior compared to the raw materials. The physical mixture of LP-PF-127 showed an endotherm at approximately 60 °C, similar to microwave processed samples (Figure 6). However, the LP-PF-127 solid dispersion itself displayed a broader, lower temperature endotherm centered around 55 °C. Both LP-PF-127 and LP-HPMC formulations presented two diffuse peaks, around 84 °C and 126 °C for the former, and 91 °C and 128 °C for the latter. Notably, the characteristic melting peak for lansoprazole was again absent in the DSC curves of the lyophilized solid dispersions.

### 3.4. PXRD

**Figure 7:**
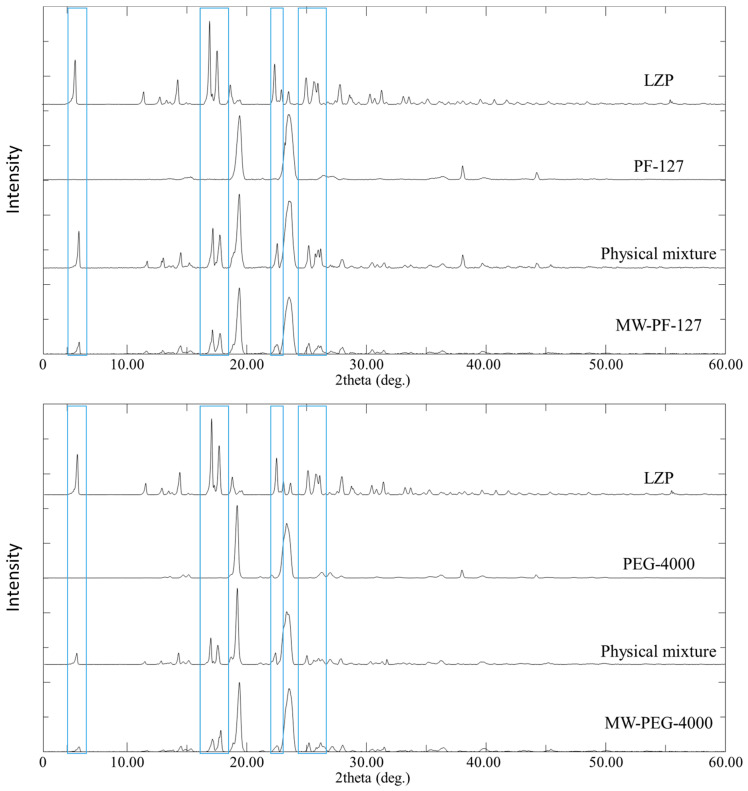
PXRD patterns of LZP, PF-127, PEG 4000, physical mixtures, and the prepared solid dispersion formulations using the microwave method. The Figure employs blue rectangles to visually designate the locations of LNS characteristic peaks of interest.

**Figure 8:**
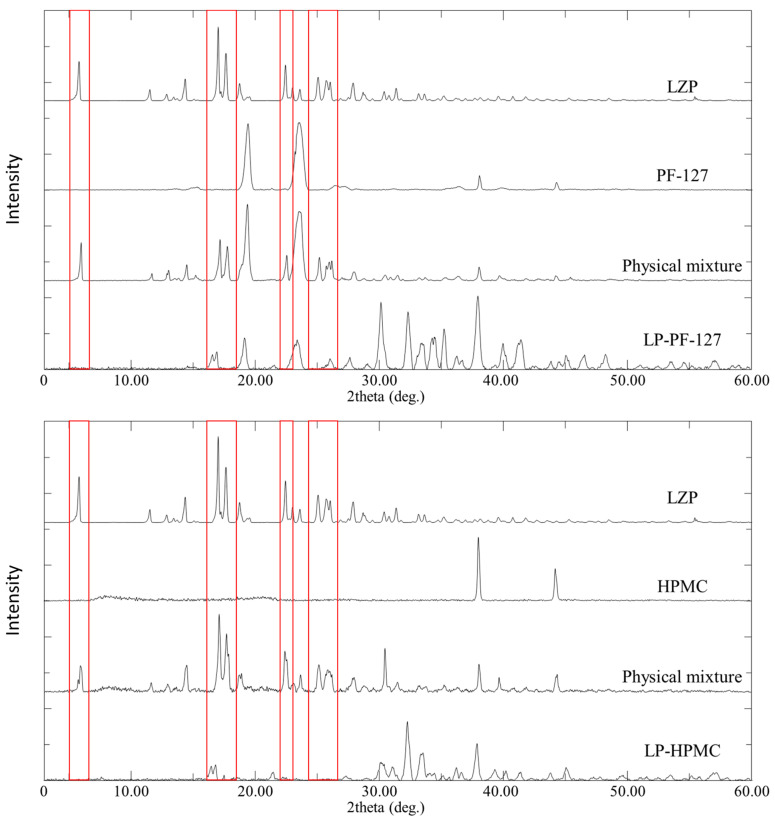
PXRD patterns of LZP, PF-127, HPMC, physical mixtures, and the prepared solid dispersion formulations using the lyophilization method. The Figure employs red rectangles to visually designate the locations of LNS characteristic peaks of interest.

The PXRD patterns of lansoprazole (LZP) exhibited characteristic peaks at 5.8, 17.0, 17.6, 22.4°, and 25–26° (Figure 7 and Figure 8). The polymers Pluronic F-127 and PEG-4000 showed peaks near 19° and 23° (Figure 7), while hydroxypropyl methylcellulose (HPMC) displayed peaks at 38.0° and 44.3° (Figure 8). As expected, physical mixtures of LZP with the polymers revealed a combination of the drug and polymer peaks (Figure 7 and Figure 8).

For microwave solid dispersions (MW-SDs), the intensities of the LZP peaks decreased but were still present, indicating some residual crystallinity. In contrast, the lyophilized SDs showed complete disappearance of the LZP peaks at 5.8° and 22.4° and a substantial reduction in the peaks at 17.0° and 17.6°. Additional peaks between 30 and 40° were observed for the lyophilized SDs.

Overall, the results suggest that the lyophilization process was more effective at disrupting the crystallinity of LZP compared to microwave irradiation. However, some residual crystalline drug was detected in both MW-SDs and lyophilized SDs by PXRD.

### 3.5. In Vitro Dissolution Studies

#### 3.5.1. SNEDDS Formulations

**Figure 9:**
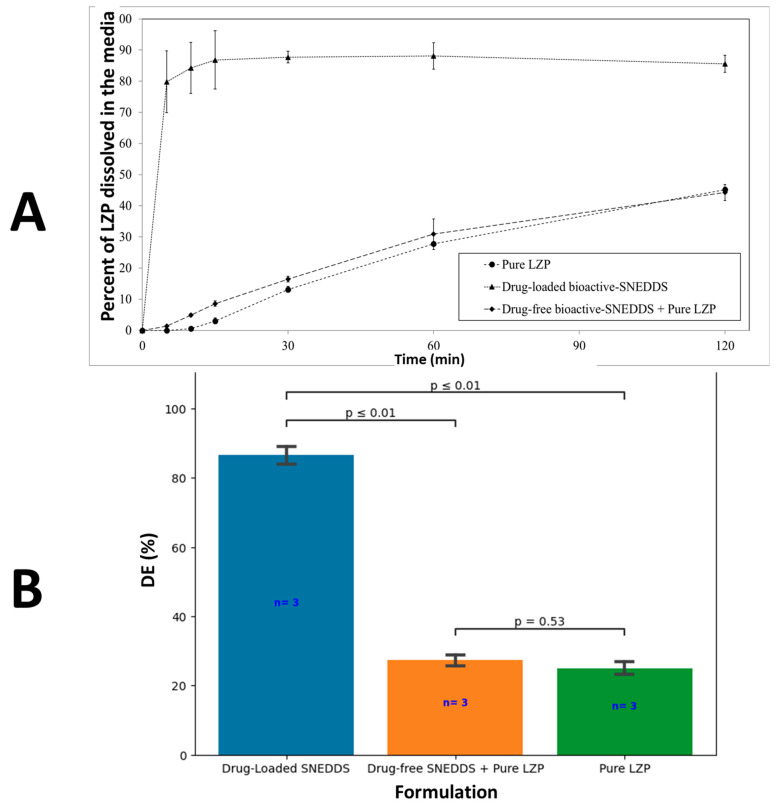
(**A**) In vitro dissolution profile of pure LZP, drug-loaded bioactive SNEDDS, and drug-free bioactive SNEDDS + pure LZP. (**B**) Graphical representation of DE of corresponding formulations.

Pure lansoprazole (LZP) exhibited poor dissolution with only 45% drug release and 25% dissolution efficiency (DE) by the end of the experiment (Figure 9A,B). In contrast, the LZP-loaded SNEDDS formulation significantly (*p* < 0.05) enhanced LZP dissolution, increasing the DE over 3-fold compared to the pure drug (Figure 9B). However, the combination of pure LZP and drug-free SNEDDS (in separate capsules) failed to improve LZP dissolution.

#### 3.5.2. SD Formulation

**Figure 10:**
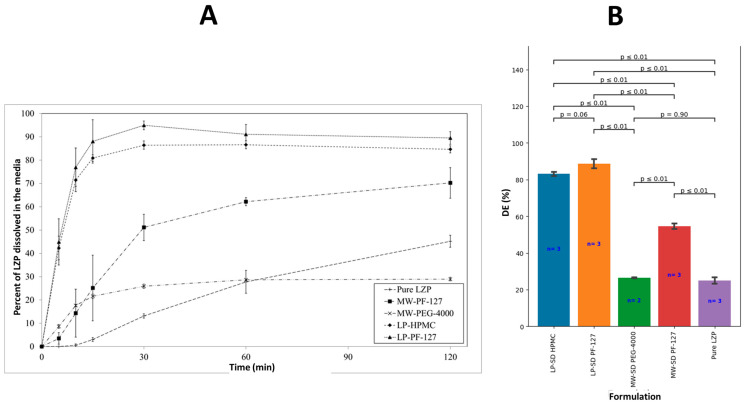
(**A**) In vitro dissolution profile of pure LZP and the prepared SD formulations. (**B**) Graphical representation of DE of corresponding formulations.

All solid dispersions significantly (*p* < 0.05) enhanced LZP release except for MW-PEG-4000 that showed slow drug release and a similar DE as the pure drug (Figure 10A,B). In particular, both LP-SD formulations (prepared using the lyophilization method) showed fast LZP release and a significantly (*p* < 0.05) higher DE compared to the MW-SD formulations. Notably, both lyophilized SDs (LP-SDs) displayed rapid drug release with a 3.5- and 3.3-fold higher DE than pure LZP for LP-PF127 and LP-HPMC, respectively.

#### 3.5.3. SD Formulation + Drug-Free Bioactive SNEDDS

**Figure 11:**
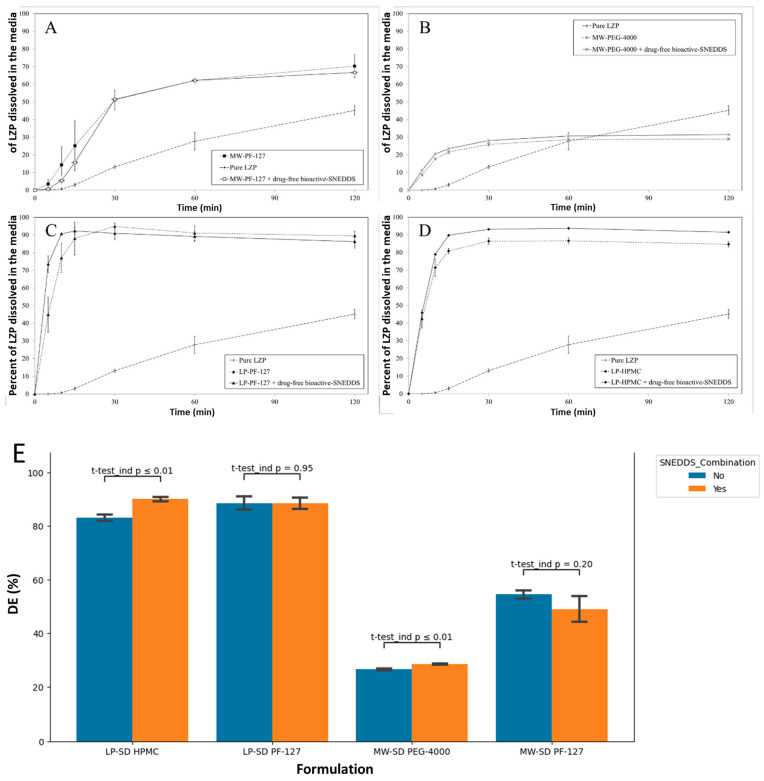
In vitro dissolution profile of pure LZP, SD formulation, and SD formulation + drug-free bioactive SNEDDS (**A**) MW-PF-127, (**B**) MW-PEG-4000, (**C**) LP-PF-127, (**D**) LP-HPMC and (**E**) Graphical representation of DE of corresponding formulations.

The two-way ANOVA test indicates that combining SDs with drug-free SNEDDS did not significantly impact DE overall (*p* = 0.51). However, the differential analysis of each formulation showed that MW-PEG-4000 and LP-HPMC SDs showed a significantly increased DE upon SNEDDS addition (*p* < 0.01) (Figure 11A,B). Meanwhile, MW-PF-127 and LP-PF-127 SDs exhibited no DE enhancement with the SNEDDS.

### 3.6. Stability Study

**Figure 12:**
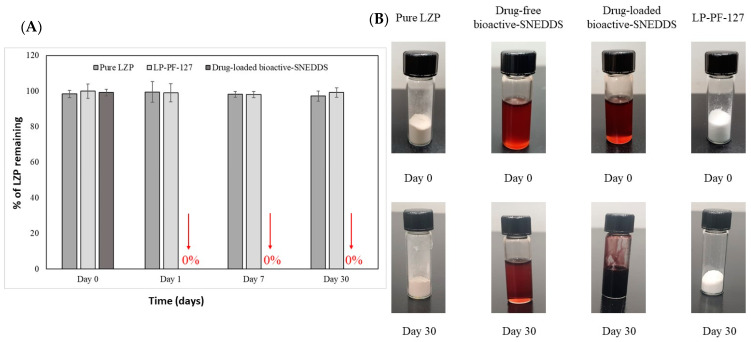
The (**A**) chemical and (**B**) physical stability study of pure LZP, LP-PF-127, and drug-loaded bioactive SNEDDS under accelerated storage conditions for 1 month (left).

The chemical stability study revealed interesting differences between the formulations. The lansoprazole (LZP)-loaded SNEDDS experienced complete drug degradation after just 1 day of storage under accelerated conditions (Figure 12A). In stark contrast, both pure LZP and the lyophilized solid dispersion LP-PF127 maintained exceptional stability, with >97% of intact drug remaining after 1 month.

Aligning with the chemical stability results, the LZP-loaded SNEDDS exhibited significant discoloration to a deep brown/black color by the end of the storage period. However, pure LZP, LP-PF127, and the drug-free SNEDDS showed no noticeable change in physical appearance after storage.

Overall, the findings indicate that the SNEDDS system afforded limited protection against drug degradation, while the lyophilized solid dispersion provided excellent protection against degradation under accelerated conditions. The physical discoloration of the SNEDDS correlates with the extensive drug degradation observed.

## 4. Discussion

In the present study, an SNEDDS was utilized to enhance the dissolution of LZP based on its previously reported advantages [34,35]. SNEDDS formulations consist of surfactants, cosurfactants, and oils, with the oils able to be derived from natural sources. Recently, there has been interest in incorporating natural compounds with pharmacological activity into SNEDDSs to provide additional therapeutic effects for diseases such as cancer [36,37], hypertension [25], and bacterial infections [38].

Previous studies have shown that thymoquinone (TMQ), a major constituent of black seed oil (BSO), possesses gastroprotective effects and can help treat peptic ulcers more potently when formulated using a SNEDDS-based delivery approach [17]. Additional research found BSO to be more effective than TMQ alone for reducing peptic ulcer indices in animal models [14]. In light of these findings, the current study selected BSO rather than isolated TMQ to develop a “bioactive-SNEDDS” system combining the benefits of the gastro-protective components intrinsic to BSO. It was hypothesized that incorporating BSO as the oil phase may augment the anti-ulcer activity of encapsulated lansoprazole (LZP). Therefore, BSO was employed not only for SNEDDS preparation but also in developing a multifunctional SNEDDS intended to co-deliver LZP and bioactive molecules from BSO which could potentially improve peptic ulcer treatment outcomes.

In the current study, the in vitro dispersion of three systems (Figure 2) were in the nanosize range which could significantly enhance LZP bioavailability following oral administration [39]. Furthermore, all the formulations exhibited negative zeta potential values ranging from −22.5 to −39 mV, which aligns with prior studies [24,40,41]. Specifically, the drug-free SNEDDS produced the highest magnitude potentials, signaling greater colloidal stability likely due to nonionic surfactant inclusion and anionic species binding to droplets. Previous research suggested that hydroxyl ions from water or fatty acid impurities in surfactants could attribute to the observed negative ZP values [42,43]. The increase in zeta potential values after incorporating the drug and solid dispersion (SD) may be linked to residues of lansoprazole and sodium carbonate from the SD, potentially introducing partial positive charges to the surface.

Furthermore, the in vitro dissolution study revealed that the prepared bioactive SNEDDS was able to increase the dissolution efficiency of LZP by 3.5-fold compared to the pure drug. However, the stability study revealed complete drug degradation following just 1 day of incubation in the stability cabinet. This could be attributed to the presence of free fatty acids in the SNEDDS components, which can create an acidic microenvironment promoting LZP degradation during storage, as reported previously [44]. Based on previous experience, the instability issues with SNEDDSs are often addressed by separating the drug from the formulation [21].

Therefore, in the current work, drug-free bioactive SNEDDS and pure LZP were placed in separate capsules and subjected to in vitro dissolution testing. However, this failed to improve the dissolution of pure LZP. Consequently, there is a need to investigate an adjuvant technology to enhance LZP dissolution alongside the drug-free bioactive SNEDDS.

Solid dispersions (SDs) were explored in this study as a means to improve LZP dissolution when co-administered with the drug-free bioactive SNEDDS, as reported previously [21,45]. For MW-SDs, LZP and the polymer (Pluronic F-127 or PEG 4000) were mixed and subjected to microwave irradiation. For the LP-SD, the drug and polymer are typically dissolved in a solvent before freeze-drying. However, the poor aqueous solubility of LZP would require a large volume of solvent for industrial production [46]. Therefore, a bicarbonate buffer was utilized as the solution medium due to its known high solubility for LZP [47] and added advantages regarding stability [48]. Accordingly, the bicarbonate buffer enabled the preparation of an LZP solution for lyophilization using a minimal solvent volume.

The DSC analyses provided vital information corroborating the changes to LZP’s physical state induced by SD preparation. The DSC spectrum of raw LZP was consistent with those previously reported in the literature, confirming the purity of the drug substance used [24,49]. In the SDs prepared by lyophilization, two new broad endothermic peaks were observed, which could have resulted from the melting of sodium carbonate and sodium bicarbonate components within the buffer system. The literature indicates that the melting points are approximately 96 °C and 160 °C for these species, respectively [50,51]. Interestingly, both MW- and LP-SDs demonstrated a disappearance of the LZP melting endotherm. This may have been due to either a dilutional effect or the transformation of the drug into an amorphous state within the polymer matrices. Therefore, PXRD analysis was subsequently conducted to further characterize the polymorphism changes in the SD formulations.

The PXRD analysis revealed changes to LZP’s crystalline state within the SD matrices prepared by the MW and LP methods. The analysis by PXRD revealed that lansoprazole (LZP) was partially or extensively converted to an amorphous form within the solid dispersion (SD) matrices prepared by the microwave (MW) and lyophilization (LP) methods. In particular, the intensities of LZP’s characteristic diffraction peaks were substantially decreased following the LP process, more so than with the MW preparation. This suggests that greater solubilization and amorphization of LZP occurred when it was dissolved within the alkaline carbonate buffer system during lyophilization, compared to the drug potentially maintaining some degree of crystallinity when subjected to microwave irradiation alone. Additionally, new peaks detected in the LP-SDs could be correlated to the presence of bicarbonate buffer components, consistent with previous reports [52,53]. These findings indicate that lyophilization more potently disrupted LZP crystallinity versus microwave heating, likely attributable to enhanced drug solubilization facilitated by the carbonate vehicle during freeze-drying.

In vitro dissolution testing was used to characterize the drug release behavior from the SDs to assess the impact of the preparation method and combination with the drug-free SNEDDS. Statistical analysis revealed that all SDs, except MW-PEG-4000, enhanced lansoprazole (LZP) dissolution relative to the pure drug. This aligns with a prior report showing a slower drug release from microwave-prepared SDs with PEG versus other polymers [54]. Additional studies found that PEG 4000 SDs enabled retardation of drug release compared to the free drug [55] and exhibited a small water absorption and solubility enhancement [56]. The inferior performance of MW-SD PEG-4000 may relate to drug recrystallization during the cooling step, as reported by Hempel et al. [57].

In contrast, the MW-PF127 SD provided a moderate ~2-fold increase in LZP dissolution efficiency, likely attributable to drug amorphization within the matrix as evidenced by PXRD. However, the lyophilized LP-PF127 SD achieved a 3.5-fold enhancement, which can be rationalized by the higher extent of drug amorphization induced during lyophilization along with the alkaline microenvironment generated by the bicarbonate buffer to facilitate wetting and dissolution. A comparable trend was observed with LP-HPMC SDs.

Combining the SDs with the drug-free bioactive SNEDDS showed no significant overall improvement in LZP release. However, MW-PEG-4000 SDs exhibited enhanced dissolution upon SNEDDS addition, potentially due to increased water absorption and wetting enabled by the self-emulsifying system. In contrast, the high water solubility and amphiphilic properties of Pluronic polymers may negate the need for an adjuvant SNEDDS to achieve optimal LZP release from PF127-based SDs [26].

LP-PF-127 exhibited the highest independent dissolution efficiency and stability, suggesting its optimization as a standalone formulation. However, incorporating the drug-free SNEDDS remains justifiable due to potential synergistic anti-ulcer effects from BSO bioactives.

There are several potential avenues for furthering this research. In vivo pharmacokinetic and pharmacodynamic studies evaluating the optimized LP-PF-127 SD-SNEDDS system could provide insights into its translation potential by comparing its performance to commercial products and other test formulations. Additional investigations into the direct gastroprotective effects of the combined drug-free SNEDDS using relevant animal disease models would help validate the hypothesized synergistic benefits from BSO bioactives. Finally, mechanistic studies examining the mode of anti-ulcer action of BSO components and how formulation impacts tissue distribution and bioavailability could provide insights to support platform translation.

## 5. Conclusions

This work demonstrates the development of an integrated gastroprotective oral delivery system containing lansoprazole solid dispersions and a bioactive black seed oil-based SNEDDS. An SNEDDS incorporating black seed oil provided a 3.5-fold improvement in lansoprazole dissolution; however, stability issues with drug loading prompted separation into a drug-free system. SD characterization revealed varying degrees of crystallinity disruption induced by different preparation techniques and polymers. In vitro dissolution directly correlated to these physicochemical property alterations, with lyophilization generating the optimal amorphous form that dramatically boosted drug release performance. Remarkably, the lyophilized Pluronic F-127 SD formulation demonstrated superior performance, and up to 97% of the drug remained stable under accelerated conditions for a month. Combining lyophilized dispersions with the bioactive SNEDDS offered minimal additional dissolution enhancement but preserved synergistic anti-ulcer potential through its natural oil components. The integrated solid dispersion–SNEDDS formulation is a promising candidate to enhance oral treatment of acid-related gastrointestinal disorders. Future work should evaluate the in vivo performance and continue optimizing the formulation to advance toward an efficacious clinical product.

## Figures and Tables

**Table 1:** Composition of solid dispersion formulations.

Formulation Code	LZP	PF-127	PEG-4000	HPMC	Sodium Hydrogen Carbonate (NaHCO_3_)	Disodium Carbonate (Na_2_CO_3_)
MW-PF-127	1.0	4.0	-	-	-	-
MW-PEG 4000	1.0	-	4.0	-	-	-
LP-PF-127	1.0	4.0	-	-	1.4	12.6
LP-HPMC	1.0	-	-	4.0	1.4	12.6

All numbers in table are expressed as mass ratios (*w*/*w*). LZP (lansoprazole), PF-127 (Pluronic-F127), PEG-4000 (polyethylene glycol-4000), HPMC (hydroxypropyl methylcellulose), MW-PF-127 (solid dispersion prepared using Pluronic-F127 by microwave method), MW-PEG-4000 (solid dispersion prepared using polyethylene glycol 4000 by microwave method), LP-PF-127 (solid dispersion prepared using Pluronic-F127 by lyophilization method), and LP-HPMC (solid dispersion prepared using hydroxypropyl methylcellulose by lyophilization method).

## Data Availability

The data presented in this study are available within the article.

## References

[1] Lien H.-M., Wang Y.-Y., Huang M.-Z., Wu H.-Y., Huang C.-L., Chen C.-C., Hung S.-W., Chen C.-C., Chiu C.-H., Lai C.-H. (2022). Gastroprotective Effect of *Anisomeles indica* on Aspirin-Induced Gastric Ulcer in Mice. Antioxidants.

[2] Martinsen T.C., Fossmark R., Waldum H.L. (2019). The phylogeny and biological function of gastric juice—Microbiological consequences of removing gastric acid. Int. J. Mol. Sci..

[3] Tolbert K., Gould E. (2020). Gastritis and gastric ulceration in dogs and cats. Clinical Small Animal Internal Medicine.

[4] Vemulapalli R. (2008). Diet and lifestyle modifications in the management of gastroesophageal reflux disease. Nutr. Clin. Pract..

[5] Kinoshita Y., Miwa H., Sanada K., Miyata K., Haruma K. (2014). Clinical characteristics and effectiveness of lansoprazole in Japanese patients with gastroesophageal reflux disease and dyspepsia. J. Gastroenterol..

[6] Srebro J., Brniak W., Mendyk A. (2022). Formulation of dosage forms with proton pump inhibitors: State of the art, challenges and future perspectives. Pharmaceutics.

[7] Lu Y., Guo T., Qi J., Zhang J., Wu W. (2012). Enhanced dissolution and stability of lansoprazole by cyclodextrin inclusion complexation: Preparation, characterization, and molecular modeling. AAPS PharmSciTech.

[8] Zhang X., Sun N., Wu B., Lu Y., Guan T., Wu W. (2008). Physical characterization of lansoprazole/PVP solid dispersion prepared by fluid-bed coating technique. Powder Technol..

[9] Fang Y., Wang G., Zhang R., Liu Z., Liu Z., Wu X., Cao D. (2014). Eudragit L/HPMCAS blend enteric-coated lansoprazole pellets: Enhanced drug stability and oral bioavailability. AAPS PharmSciTech.

[10] Alsulays B.B., Kulkarni V., Alshehri S.M., Almutairy B.K., Ashour E.A., Morott J.T., Alshetaili A.S., Park J.-B., Tiwari R.V., Repka M.A. (2017). Preparation and evaluation of enteric coated tablets of hot-melt extruded lansoprazole. Drug Dev. Ind. Pharm..

[11] Ubgade S., Bapat A., Kilor V. (2021). Effect of various stabilizers on the stability of lansoprazole nanosuspension prepared using high shear homogenization: Preliminary investigation. J. Appl. Pharm. Sci..

[12] Morakul B. (2020). Self-nanoemulsifying drug delivery systems (SNEDDS): An advancement technology for oral drug delivery. Pharm. Sci. Asia.

[13] Kazi M., Shahba A.A., Alrashoud S., Alwadei M., Sherif A.Y., Alanazi F.K. (2020). Bioactive self-nanoemulsifying drug delivery systems (Bio-SNEDDS) for combined oral delivery of curcumin and piperine. Molecules.

[14] Kanter M., Coskun O., Uysal H. (2006). The antioxidative and antihistaminic effect of *Nigella sativa* and its major constituent, thymoquinone on ethanol-induced gastric mucosal damage. Arch. Toxicol..

[15] Jarmakiewicz-Czaja S., Zielińska M., Helma K., Sokal A., Filip R. (2023). Effect of *Nigella sativa* on Selected Gastrointestinal Diseases. Curr. Issues Mol. Biol..

[16] Zeren S., Bayhan Z., Kocak F.E., Kocak C., Akcılar R., Bayat Z., Simsek H., Duzgun S.A. (2016). Gastroprotective effects of sulforaphane and thymoquinone against acetylsalicylic acid–induced gastric ulcer in rats. J. Surg. Res..

[17] Radwan M.F., El-Moselhy M.A., Alarif W.M., Orif M., Alruwaili N.K., Alhakamy N.A. (2021). Optimization of Thymoquinone-Loaded Self-Nanoemulsion for Management of Indomethacin-Induced Ulcer. Dose-Response.

[18] Paseban M., Niazmand S., Soukhtanloo M., Meibodi N.T., Abbasnezhad A., Mousavi S.M., Niazmand M.J. (2020). The therapeutic effect of *Nigella sativa* seed on indomethacin-induced gastric ulcer in Rats. Curr. Nutr. Food Sci..

[19] Bethesda (MD): National Institute of Diabetes and Digestive and Kidney Diseases; 2012-. LiverTox: Clinical and Research Information on Drug-Induced Liver Injury [Internet]. Black Cumin Seed. [Updated 2023 Apr 27]. https://www.ncbi.nlm.nih.gov/books/NBK591552/.

[20] Ashfaq M., Shah S., Rasul A., Hanif M., Khan H.U., Khames A., Abdelgawad M.A., Ghoneim M.M., Ali M.Y., Abourehab M.A. (2022). Enhancement of the solubility and bioavailability of pitavastatin through a self-nanoemulsifying drug delivery system (SNEDDS). Pharmaceutics.

[21] Shahba A.A., Tashish A.Y., Alanazi F.K., Kazi M. (2021). Combined Self-Nanoemulsifying and Solid Dispersion Systems Showed Enhanced Cinnarizine Release in Hypochlorhydria/Achlorhydria Dissolution Model. Pharmaceutics.

[22] Shahba A.A.-W., Ahmed A.R., Alanazi F.K., Mohsin K., Abdel-Rahman S.I. (2018). Multi-layer self-nanoemulsifying pellets: An innovative drug delivery system for the poorly water-soluble drug cinnarizine. AAPS PharmSciTech.

[23] Nair A.R., Lakshman Y.D., Anand V.S.K., Sree K.N., Bhat K., Dengale S.J. (2020). Overview of extensively employed polymeric carriers in solid dispersion technology. AAPS PharmSciTech.

[24] Alshadidi A., Shahba A.A., Sales I., Rashid M.A., Kazi M. (2021). Combined Curcumin and Lansoprazole-Loaded Bioactive Solid Self-Nanoemulsifying Drug Delivery Systems (Bio-SSNEDDS). Pharmaceutics.

[25] Shahba A.A.-W., Sherif A.Y., Elzayat E.M., Kazi M. (2022). Combined Ramipril and Black Seed Oil Dosage Forms Using Bioactive Self-Nanoemulsifying Drug Delivery Systems (BIO-SNEDDSs). Pharmaceuticals.

[26] Aboutaleb A.E., Abdel-Rahman S.I., Ahmed M.O., Younis M.A. (2016). Improvement of domperidone solubility and dissolution rate by dispersion in various hydrophilic carriers. J. Appl. Pharm. Sci..

[27] Alshehri S., Shakeel F., Ibrahim M., Elzayat E., Altamimi M., Shazly G., Mohsin K., Alkholief M., Alsulays B., Alshetaili A. (2017). Influence of the microwave technology on solid dispersions of mefenamic acid and flufenamic acid. PLoS ONE.

[28] Tashish A.Y., Shahba A.A.-W., Alanazi F.K., Kazi M. (2023). Adsorbent Precoating by Lyophilization: A Novel Green Solvent Technique to Enhance Cinnarizine Release from Solid Self-Nanoemulsifying Drug Delivery Systems (S-SNEDDS). Pharmaceutics.

[29] Tian Z., Yi Y., Yuan H., Han J., Zhang X., Xie Y., Lu Y., Qi J., Wu W. (2013). Solidification of nanostructured lipid carriers (NLCs) onto pellets by fluid-bed coating: Preparation, in vitro characterization and bioavailability in dogs. Powder Technol..

[30] El Maghraby G.M., Elzayat E.M., Alanazi F.K. (2012). Development of modified in situ gelling oral liquid sustained release formulation of dextromethorphan. Drug Dev. Ind. Pharm..

[31] Shahba A.A.-W., Alanazi F.K., Abdel-Rahman S.I. (2018). Stabilization benefits of single and multi-layer self-nanoemulsifying pellets: A poorly-water soluble model drug with hydrolytic susceptibility. PLoS ONE.

[32] Mishra P., Pandey C.M., Singh U., Gupta A., Sahu C., Keshri A. (2019). Descriptive statistics and normality tests for statistical data. Ann. Card. Anaesth..

[33] Nahm F.S. (2016). Nonparametric statistical tests for the continuous data: The basic concept and the practical use. Korean J Anesth..

[34] Rehman F.U., Shah K.U., Shah S.U., Khan I.U., Khan G.M., Khan A. (2017). From nanoemulsions to self-nanoemulsions, with recent advances in self-nanoemulsifying drug delivery systems (SNEDDS). Expert Opin. Drug Deliv..

[35] Krstić M., Medarević Đ., Đuriš J., Ibrić S. (2018). Self-nanoemulsifying drug delivery systems (SNEDDS) and self-microemulsifying drug delivery systems (SMEDDS) as lipid nanocarriers for improving dissolution rate and bioavailability of poorly soluble drugs. Lipid Nanocarriers for Drug Targeting.

[36] Kazi M., Nasr F.A., Noman O., Alharbi A., Alqahtani M.S., Alanazi F.K. (2020). Development, characterization optimization, and assessment of curcumin-loaded bioactive self-nanoemulsifying formulations and their inhibitory effects on human breast cancer MCF-7 cells. Pharmaceutics.

[37] Alwadei M., Kazi M., Alanazi F.K. (2019). Novel oral dosage regimen based on self-nanoemulsifying drug delivery systems for codelivery of phytochemicals–curcumin and thymoquinone. Saudi Pharm. J..

[38] Kazi M., Alhajri A., Alshehri S.M., Elzayat E.M., Al Meanazel O.T., Shakeel F., Noman O., Altamimi M.A., Alanazi F.K. (2020). Enhancing oral bioavailability of apigenin using a bioactive self-nanoemulsifying drug delivery system (Bio-SNEDDS): In vitro, in vivo and stability evaluations. Pharmaceutics.

[39] Pathak K., Raghuvanshi S. (2015). Oral bioavailability: Issues and solutions via nanoformulations. Clin. Pharmacokinet..

[40] Shakeel F., Haq N., El-Badry M., Alanazi F.K., Alsarra I.A. (2013). Ultra fine super self-nanoemulsifying drug delivery system (SNEDDS) enhanced solubility and dissolution of indomethacin. J. Mol. Liq..

[41] Zhao Y., Wang C., Chow A.H., Ren K., Gong T., Zhang Z., Zheng Y. (2010). Self-nanoemulsifying drug delivery system (SNEDDS) for oral delivery of Zedoary essential oil: Formulation and bioavailability studies. Int. J. Pharm..

[42] Silva H.D., Cerqueira M.A., Vicente A.A. (2015). Influence of surfactant and processing conditions in the stability of oil-in-water nanoemulsions. J. Food Eng..

[43] Tian Y., Chen L., Zhang W. (2016). Influence of Ionic Surfactants on the Properties of Nanoemulsions Emulsified by Nonionic Surfactants Span 80/Tween 80. J. Dispers. Sci. Technol..

[44] He W., Yang M., Fan J.H., Feng C.X., Zhang S.J., Wang J.X., Guan P.P., Wu W. (2010). Influences of sodium carbonate on physicochemical properties of lansoprazole in designed multiple coating pellets. AAPS PharmSciTech.

[45] Nora G.-I., Venkatasubramanian R., Strindberg S., Siqueira-Jørgensen S.D., Pagano L., Romanski F.S., Swarnakar N.K., Rades T., Müllertz A. (2022). Combining lipid based drug delivery and amorphous solid dispersions for improved oral drug absorption of a poorly water-soluble drug. J. Control. Release.

[46] Fang Z., Bhandari B. (2012). Spray drying, freeze drying and related processes for food ingredient and nutraceutical encapsulation. Encapsulation Technologies and Delivery Systems for Food Ingredients and Nutraceuticals.

[47] Tabatar T., Makino T., Kashihara T., Hirai S., Kitamori N., Toguchi H. (1992). Stabilization of a new antiulcer drug (lansoprazole) in the solid dosage forms. Drug Dev. Ind. Pharm..

[48] Pasic M. (2008). Study to Design Stable Lansoprazole Pellets. Ph.D. Thesis.

[49] Yu M., Sun L., Li W., Lan Z., Li B., Tan L., Li M., Yang X. (2011). Investigation of structure and dissolution properties of a solid dispersion of lansoprazole in polyvinylpyrrolidone. J. Mol. Struct..

[50] Harabor A., Rotaru P., Harabor N. (2013). Two phases in a commercial anhydrous sodium carbonate by air contact. Phys. AUC.

[51] Leemsuthep A., Mohd Nayan N.A., Zakaria Z., Uy Lan D.N. (2017). Effect of sodium bicarbonate in fabrication of carbon black-filled epoxy porous for conductive application. Proceedings of the Macromolecular Symposia—Special Issue: 4th Federation of Asian Polymer Societies International Polymer Congress.

[52] Uysal D., Dogan Ö.M., Uysal B.Z. (2017). Kinetics of absorption of carbon dioxide into sodium metaborate solution. Int. J. Chem. Kinet..

[53] Reddy V.V., Rao H.S., Jayaveera K. (2006). Influence of strong alkaline substances (sodium carbonate and sodium bicarbonate) in mixing water on strength and setting properties of concrete. Indian J. Eng. Mater. Sci..

[54] Alshehri S., Alanazi A., Elzayat E.M., Altamimi M.A., Imam S.S., Hussain A., Alqahtani F., Shakeel F. (2021). Formulation, in vitro and in vivo evaluation of gefitinib solid dispersions prepared using different techniques. Processes.

[55] Moneghini M., De Zordi N., Grassi M., Zingone G. (2008). Sustained-release solid dispersions of ibuprofen prepared by microwave irradiation. J. Drug Deliv. Sci. Technol..

[56] Tafu N.N., Jideani V.A. (2022). Proximate, elemental, and functional properties of novel solid dispersions of *Moringa oleifera* leaf powder. Molecules.

[57] Hempel N.-J., Knopp M.M., Zeitler J.A., Berthelsen R., Löbmann K. (2021). Microwave-induced in situ drug amorphization using a mixture of polyethylene glycol and polyvinylpyrrolidone. J. Pharm. Sci..

